# Supplementary material for: The Escherichia coli S2P intramembrane protease RseP regulates ferric citrate uptake by cleaving the sigma factor regulator FecR
Source: J Biol Chem. 2021 Apr 16;296:100673. doi: 10.1016/j.jbc.2021.100673 (PMC8144685; doi:10.1016/j.jbc.2021.100673)
Supplement: Supplemental Figures S1–S8 and Tables S2–S5 [file mmc1.pdf]

Supporting Information

The *Escherichia coli* S2P intramembrane protease RseP regulates ferric citrate uptake by cleaving the sigma factor regulator FecR

Tatsuhiko Yokoyama[1], Tomoya Niinae[2], Kazuya Tsumagari[2], Koshi Imami[2], Yasushi Ishihama[2], Yohei Hizukuri[1,*], Yoshinori Akiyama[1,*]

1, Institute for Frontier Life and Medical Sciences, Kyoto University, Kyoto 606-8507, Japan

2, Graduate School of Pharmaceutical Sciences, Kyoto University, Kyoto 606-8501, Japan

*For correspondence:

yakiyama@infront.kyoto-u.ac.jp

yhizukur@infront.kyoto-u.ac.jp

Tel. +81 75-751-4040; Fax +81 75-771-5699

Contents

1. **Supplementary Results (p. 2)**
2. **Supplementary Figures S1-S8 (p. 3-10)**
3. **Supplementary Tables S2-S5 (p. 11-14)**
4. **Supplementary Experimental Procedures (p. 15-17)**

1. Supplementary Results

We analyzed the FecR proteins by immunoblotting or pulse-chase assays in several experiments conducted by using the medium containing $FeCl_3$ (Figs. 3C, 4B, 4C, 5, 6, S2, S3 and S4). Without the addition of $FeCl_3$, we observed only a small and variable degree of F-MBP-FecR processing (data not shown), which was presumably induced by contaminated ferric ions. Examination of F-MBP-FecR processing in the Δ*rseA* Δ*rseP* strain in the presence of varying concentrations of $FeCl_3$ showed that in the presence of 1 μM or higher concentrations of $FeCl_3$, the relative amount of CL(b) was maximized and the results were highly reproducible (Fig. S8). We thus added 10 μM $FeCl_3$ to the medium to examine the processing that FecR protein receives in the experiments mentioned above.

2. Supplementary Figures

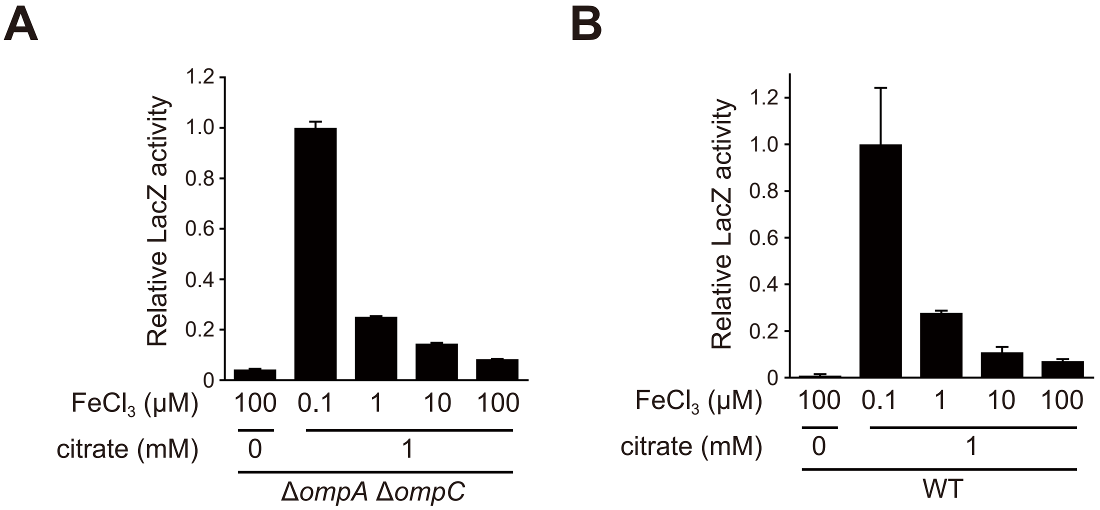

Figure S1. Responses of the promoter activity of P_{fecA} to $FeCl_3$. YH426 (Δ*ompA* Δ*ompC*) cells with pYK149 (P_{fecA}-*lacZ*) (A) and MC4100 (wild-type, WT) cells with pYK149 (P_{fecA}-*lacZ*), pSTD343 (*lacI*), and pSTD1060 (vec) (B) were grown at 30°C in M9-based medium containing the indicated concentrations of $FeCl_3$ and Na_3-citrate until mid-log phase and the LacZ activity was measured. The relative LacZ activities was normalized by the LacZ activity of the cells grown in medium containing 0.1 μM $FeCl_3$ and 1 mM Na_3-citrate are shown. Two independent experiments were carried out and the mean values are shown with standard deviations.

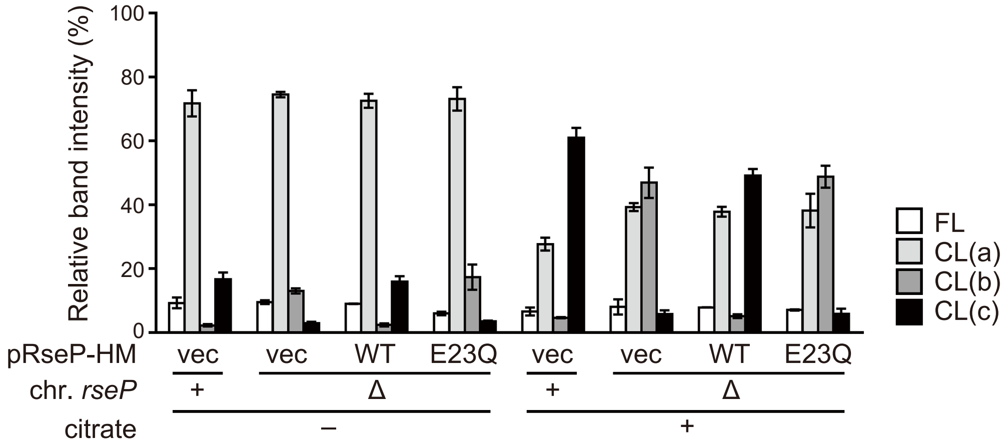

Figure S2. Quantified results of the accumulation of the processed products of FecR. Dependency of the generation of each processed product on the proteolytic activity of RseP and the addition of citrate. The band intensities of FL, CL(a), CL(b), and CL(c) derived from F-MBP-FecR in Fig. 4B and in an independently-repeated experiment were quantified. The percentages of the intensities of each band are plotted. The averages of the values are shown with standard deviations.

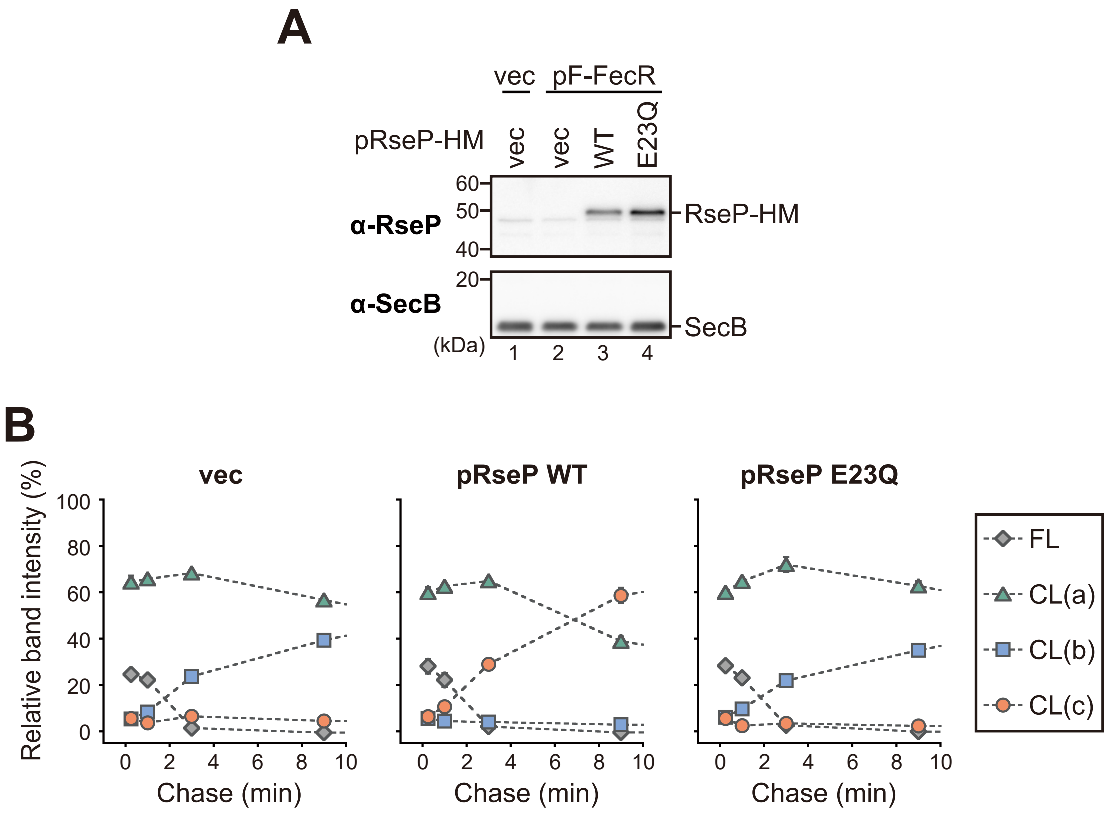

Figure S3. Accumulation of RseP and close-up views of the quantified pulse-chase results in the experiment shown in Figure 5. (A) Accumulation of RseP in the pulse-chase experiments shown in Fig. 5. The cells were grown as described in the legend to Fig. 5. Then the cultures were divided into two portions. One was induced with 1 mM IPTG and 1 mM cAMP for 10 min, and used for the pulse-chase experiment shown in Fig. 5. The other was induced with IPTG and cAMP similarly and subjected to analysis by 12.5% Laemmli SDS-PAGE and anti-RseP (α-RseP) or anti-SecB (α-SecB) immunoblotting. RseP-HM shown on the right indicates the accumulated RseP-HM or RseP(E23Q)-HM. Cytoplasmic protein SecB serves as a loading control. The positions of molecular size markers (in kDa) are shown on the left. (B) Close-up views of the 0-10 min chase period of the graphs shown in Fig. 5B.

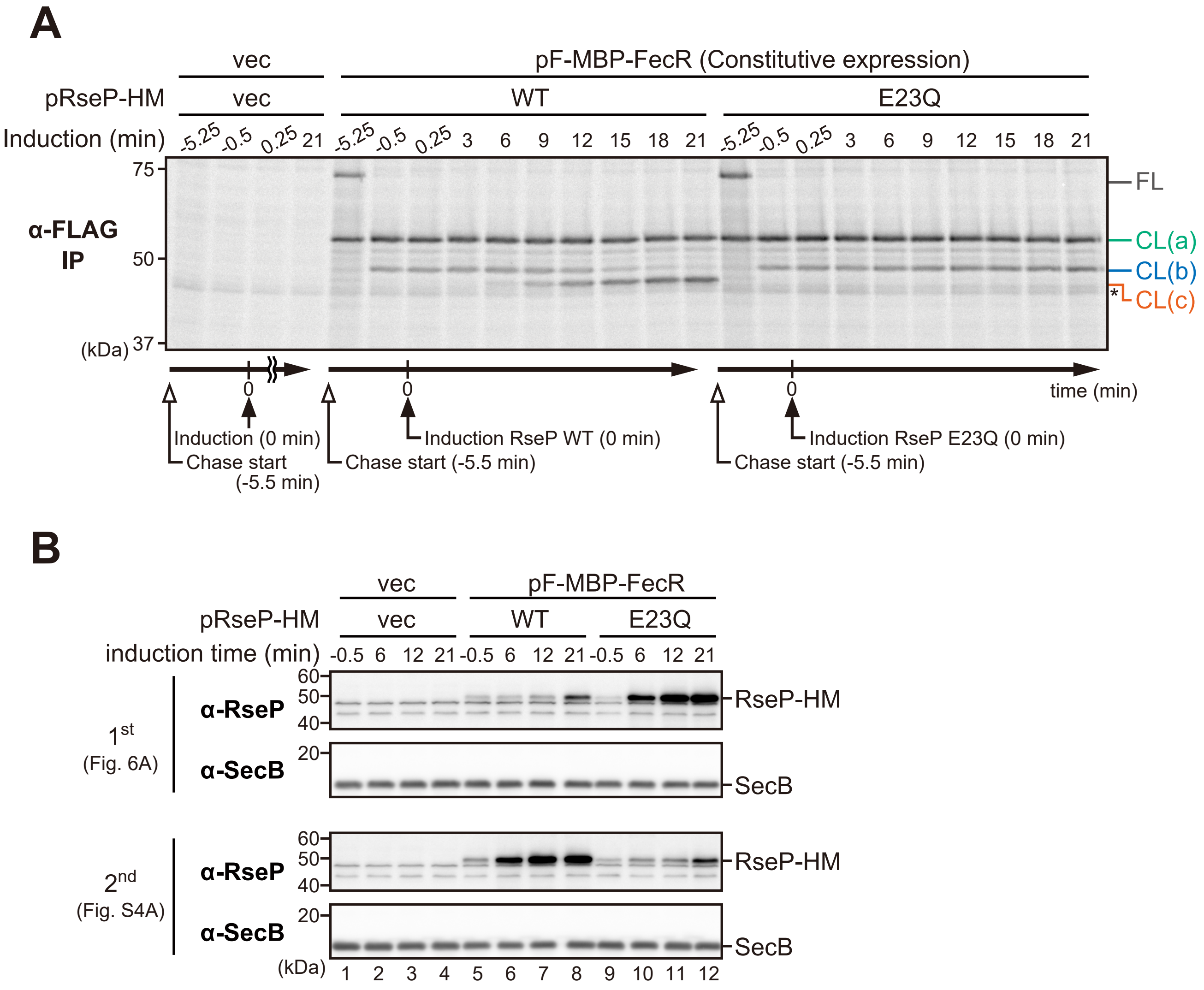

Figure S4. RseP-dependent conversion of the FecR CL(b) fragment to CL(c). (A) Pulse-chase analysis of F-MBP-FecR was conducted as Fig. 6A. (B) The RseP-accumulation in the two experiments shown in Fig. 6A and (A). The cells were grown as in Fig. 6A. Then the cultures were divided into two portions and one was used for the pulse-chase analyses as described in Fig. 6A. The other was induced with 1 mM IPTG and 1 mM cAMP for the indicated periods and the proteins were analyzed as in Fig. S3A. Note that the intensities of some of the bands for RseP-HM are saturated.

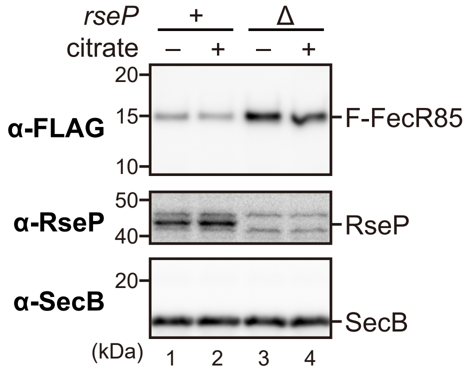

Figure S5. RseP-dependency of the behavior of F-FecR85. YK630 (Δ*rseA rseP*[+]) or YK797 (Δ*rseA* Δ*rseP*) cells harboring pYK149 (P_{fecA}-*lacZ*), pSTD343 (*lacI*), and pYK200 (pF-FecR85) were grown as in Fig. 7C and the proteins were analyzed by 15% Bis-Tris SDS-PAGE and anti-FLAG (α-FLAG) immunoblotting. Chromosomally-encoded RseP and SecB were also analyzed as in Fig. S3A.

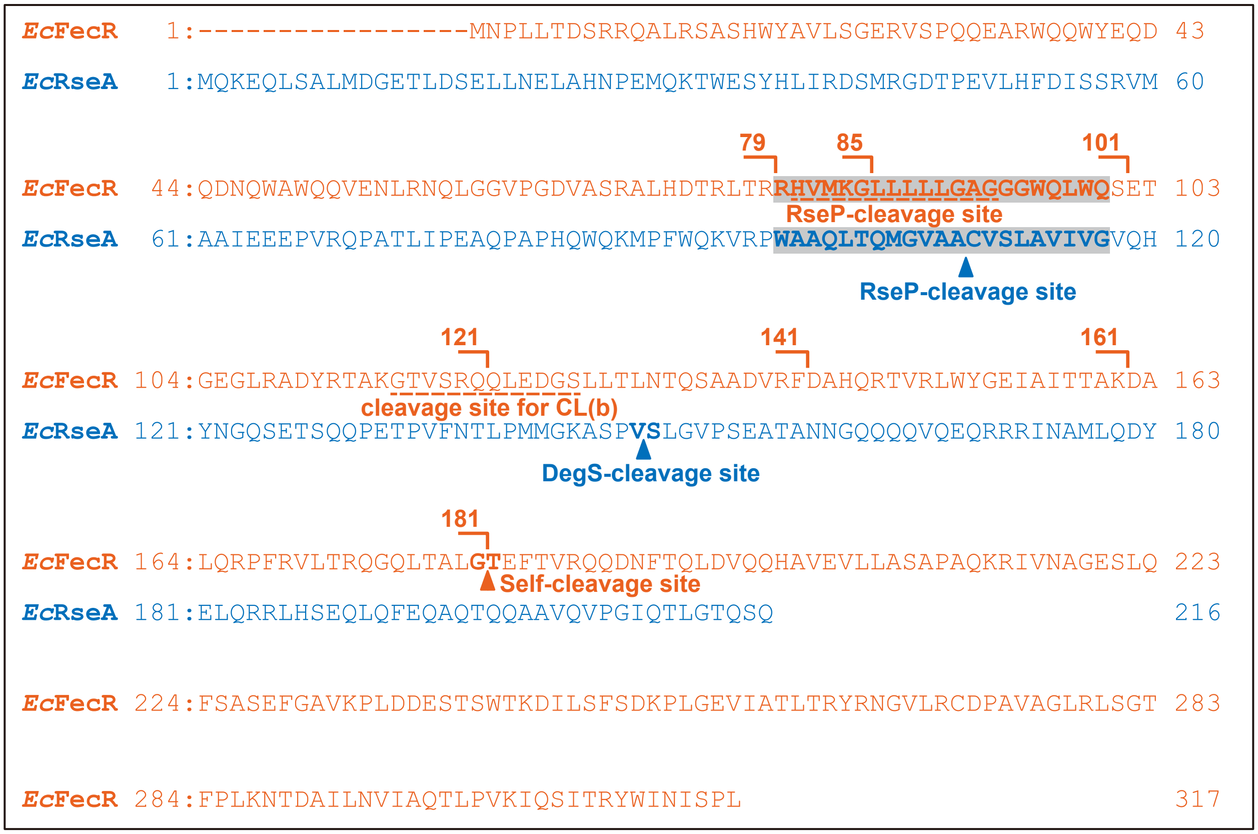

Figure S6. Sequence alignment of FecR and RseA. The amino acid sequences of *Escherichia coli* FecR (*Ec*FecR, UniProtKB accession number: P23485) and RseA (*Ec*RseA, UniProtKB accession number: P0AFX7) are aligned on the basis of the locations of the predicted transmembrane regions. The amino acid residues in the predicted transmembrane regions are shown in boldface and shaded. The positions of the C-termini of the truncated FecR mutants used in this study were shown above each sequence. Broken lines indicate the regions in which the cleavages generating CL(b) and CL(c) are expected to occur.

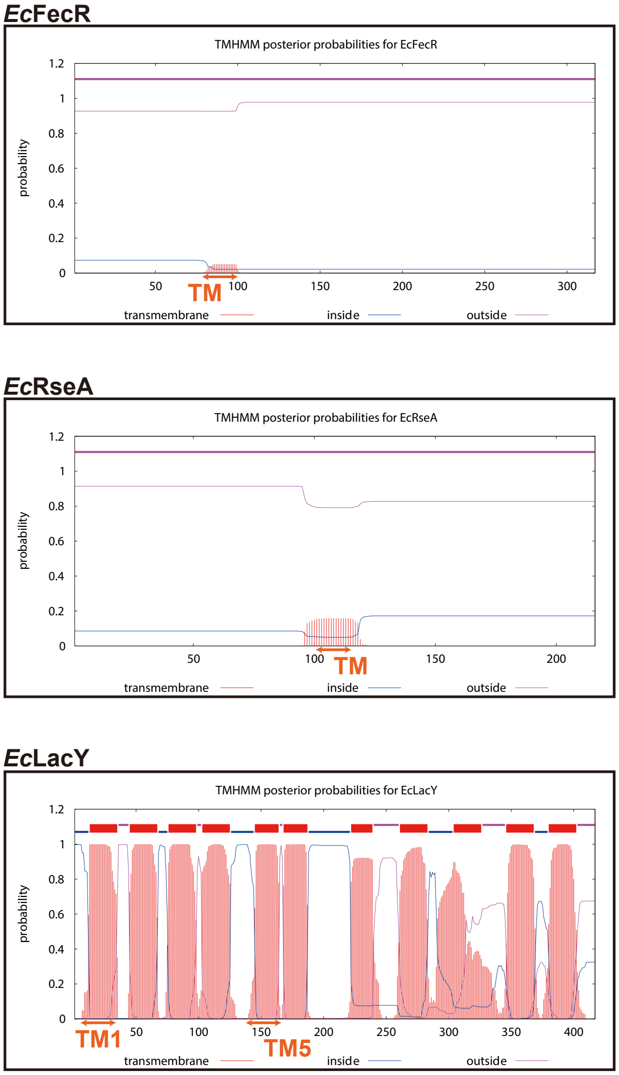

Figure S7. Prediction of the transmembrane regions in FecR, RseA and LacY by TMHMM. The transmembrane regions and membrane topologies of *Ec*FecR, *Ec*RseA and *Ec*LacY (UniProtKB accession number: P02920) were predicted by the TMHMM program (http://www.cbs.dtu.dk/services/TMHMM/) (70, 93). The transmembrane region of FecR predicted by SPOCTOPUS and the assigned transmembrane region of RseA, LacY TM1 (the first transmembrane segment) and TM5 (the fifth transmembrane segment) in Akiyama *et al*. (2004) (29) are indicated by orange bidirectional arrows.

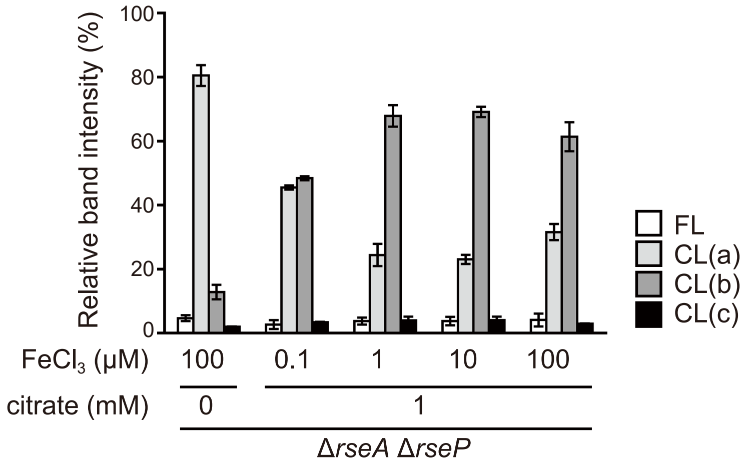

Figure S8. Response of the fragmentation of F-MBP-FecR to $FeCl_3$. Dependency of the generation of the processed product on the $FeCl_3$ and citrate concentrations in the medium. YK191 (Δ*rseA* Δ*rseP*) cells harboring pYK147 (pF-MBP-FecR) and pSTD689 (vec). The cells are grown at 30°C in M9-based medium containing 1 mM IPTG, 1 mM cAMP and the indicated concentrations of $FeCl_3$ and Na_3-citrate. Proteins were analyzed as in Fig. 4B. Quantification of the band intensities was performed as in Fig. S2.

3. Supplementary Tables

Table S2. Gene-annotation enrichment analysis by DAVID. UniProtKB keywords are listed (*p*-value < 0.05).

UP regulated

**Term**	**Number of Genes**	**Fold Enrichment**	***p*-value**
TonB box	3	36.08	2.38E-03
Receptor	3	31.57	3.16E-03
Iron transport	3	15.78	1.28E-02
Transmembrane beta strand	3	14.03	1.62E-02
Ion transport	4	5.91	2.32E-02
Cell outer membrane	3	5.26	9.81E-02
Signal	7	3.22	1.07E-02
Transmembrane	12	2.54	7.10E-04
Membrane	14	2.13	5.84E-04
Transmembrane helix	9	2.00	3.69E-02
Cell membrane	10	1.71	5.92E-02

DOWN regulated

**Term**	**Number of Genes**	**Fold Enrichment**	***p*-value**
Iron transport	3	10.82	2.94E-02
rRNA processing	3	8.65	4.43E-02
Ligase	5	5.49	1.13E-02
Acetylation	4	4.16	6.61E-02
RNA-binding	5	4.14	2.90E-02
Ion transport	4	3.67	8.89E-02
Zinc	5	2.79	9.53E-02
ATP-binding	10	2.45	1.46E-02
Nucleotide-binding	11	2.28	1.47E-02
Cytoplasm	13	2.03	1.59E-02

Table S3. Strains used in this study

Name	Genotype	Reference
MC4100	F^{-}, *araD139* Δ*(argF-lac)U169 rpsL150 relA1 flbB5301 deoC1 ptsF25 rbsR*	(94)
YK627	MC4100, Δ*fecR1*	This study
YH426	MC4100, Δ*ompA* Δ*ompC*	(28)
KA363	MC4100, Δ*ompA* Δ*ompC* Δ*rseP*::*kan*	(28)
YK630	MC4100, Δ*ompA* Δ*ompC* Δ*fecR1*	This study
YK797	MC4100, Δ*ompA* Δ*ompC* Δ*fecR1* Δ*rseP*::*kan*	This study
CU141	MC4100, /F' *lacI*^{q} *Z*$^{+}$ *Y*$^{+}$	(95)
KK377	CU141, Δ*rseA*::*cat* Δ*rseP*::*kan*	(29)
HM1742	CU141, *ara*$^{+}$	(96)
YK167	HM1742, Δ*rseA*	This study
YK191	HM1742, Δ*rseA* Δ*rseP*::*kan*	This study
BW25113	F^{-}, *rrnB* Δ*lacZ4787 hsdR514* Δ(*araBAD*)*567* Δ(*rhaBAD*)*568 rph-1*	(97)
YK602	BW25113, Δ*fecR1*::*kan*	This study
JW2556	BW25113, Δ*rseA*::*kan,* KEIO collection	(98)
AD16	Δ*pro-lac thi* /F' *lacI*^{q} *Z*ΔM15 *Y*$^{+}$ *pro*$^{+}$	(99)
AD1840	AD16, Δ*rseA*::*cat* Δ*rseP*::*kan* Δ*degS*::*tet*	(9)

Table S4. Plasmids used in this study

Name	Vector	Encoded proteins or descriptions	Reference
pBAD33		pACYC184-based vector; P_{BAD}, Cm^R	(100)
pTWV228		pBR322-based vector; P_{lac}, Amp^R	Takara Bio
pMW118		pSC101-based vector; P_{lac}, Amp^R	Nippon Gene
pSTV29		pACYC184-based vector; P_{lac}, Cm^R	Takara Bio
pSTD689		pACYC184-based vector; P_{lac}, Spc^R	(101)
pFZY1		F-based vector; LacZ-reporter plasmid; Ap^R	(102)
pHP45Ω		Spc^R coding vector	(103)
pSTD1060		pBR322-based vector; P_{lac}, Spc^R	This study
pYH604		pTWV228-based constitutive expression vector	This study
pKD13		Template for the PCR of cassette construction	(97)
pKD46		λ-Red recombinase system	(97)
pCP20		FLP recombinase	(104)
pKK6	pBAD33	RseP	(10)
pKK47	pTWV228	RseP	A gift of Kanehara Kazue
pYK2	pTWV228	RseP(E23Q)	This study
pYH9	pSTD689	RseP-His_6-Myc	(82)
pYH13	pSTD689	RseP(E23Q)-His_6-Myc	(82)
pYK151	pSTD1060	RseP-His_6-Myc	This study
pYK153	pSTD1060	RseP(E23Q)-His_6-Myc	This study
pYK124	pTWV228	FecR	This study
pYK186	pSTD1060	FecR	This study
pYK198	pSTD1060	FecR85	This study
pYK126	pTWV228	HA-FecR	This study
pYK130	pTWV228	3xFLAG-FecR	This study
pYK188	pSTD1060	3xFLAG-FecR	This study
pYK200	pSTD1060	3xFLAG-FecR85	This study
pYH19	pTWV228	HA-MBP-RseA148	(82)
pYK143	pTWV228	3xFLAG-MBP-RseA148	This study
pYK147	pTWV228	3xFLAG-MBP-FecR	This study
pYK172	pTWV228	3xFLAG-MBP-FecR181	This study
pYK170	pTWV228	3xFLAG-MBP-FecR161	This study
pYK168	pTWV228	3xFLAG-MBP-FecR141	This study
pYK166	pTWV228	3xFLAG-MBP-FecR121	This study
pYK164	pTWV228	3xFLAG-MBP-FecR101	This study
pYK174	pTWV228	3xFLAG-MBP-FecR85	This study
pYK162	pTWV228	3xFLAG-MBP-FecR79	This study
pYK182	pYH604	3xFLAG-MBP-FecR	This study
pYK190	pSTD1060	3xFLAG-MBP-FecR	This study
pYK140	pTWV228	Promoter of *fecA*	This study
pYK145	pFZY1	P_{fecA}-*lacZ*	This study
pYK149	pMW118	P_{fecA}-*lacZ*	This study
pSTD343	pSTV29	*lacI*	(105)

Amp^R, ampicillin-resistance marker; Spc^R, spectinomycin-resistance marker; Km^R, kanamycin-resistance marker; Cm^R, chloramphenicol-resistance marker.

Table S5. Oligonucleotides used in this study

Number	Name	Sequence
P1	fecR_disrupt(p)	TGCTGTTCCGTCTGGAGTATGGGTTATGAATCCTTTGTTA GTGTAGGCTGGAGCTGCTTC
P2	fecR_disrupt(m)	ACAATCGAAATAAGAATTATTTTCCTTACAGTGGTGAAAT ATTCCGGGGATCCGTCGACC
P3	SacI-SD-fecR(p)	TACGCGAGCTCGGTACAAGGAGGAAGAGCAAATGAATC CTTTGTTAACCGATTC
P4	KpnI-fecR(p)	TACGCGGTACCCAATCCTTTGTTAACCGATTCCCG
P5	BamHI-fecR TM(p)	TACGCGGATCCGATACCCGCCTCACCCGCCGTC
P6	fecR-HindIII(m)	GCGTAAAGCTTTTACAGTGGTGAAATGTTTATCC
P7	Oligo(p) SacI-3xFLAG-KpnI	CGGTACAAGGAGGAAGAGCAAATGGACTACAAAGACCA TGACGGTGATTATAAAGATCATGACATCGACTACAAAGA CGATGACGACAAGTCGGTAC
P8	Oligo(m) SacI-3xFLAG-KpnI(-)	CGACTTGTCGTCATCGTCTTTGTAGTCGATGTCATGATCT TTATAATCACCGTCATGGTCTTTGTAGTCCATTTGCTCTTC CTCCTTGTACCGAGCT
P9	KpnI-PfecA(p)	CGGGGTACCCGCTTCCCGTTAAAATTCAGTC
P10	PfecA-BamHI(m)	CGCGGATCCCCATCATCATTTTTGTTGTGTTC
P11	llinepMW118(p)	CATTGTCGATCTGTTCATGGTGA
P12	linepMW118(m)	TCGGCCAACGCGCGGGGA
P13	insViapYK145(p)	CCGCGCGTTGGCCGAGAATTCGAGCTCGGTACCC
P14	insViapYK145(m)	AACAGATCGACAATGTTATTTTGACACCAGACCAACTG

4. Supplementary Experimental Procedures

Construction of strains

YK167 was constructed as follows. The Δ*rseA*::*kan* region from JW2556 (98) was introduced into HM1742 by P1 transduction, and the *kan* cassette of the resulting strain was deleted using pCP20, as described previously (97). YK191 was constructed by transferring the Δ*rseP*::*kan* marker from KK377 into YK167 by P1 transduction. YK602 (Δ*fecR1*::*kan*) was constructed by deleting the chromosomal *fecR* gene using the one-step method described by Datsenko and Wanner (2000) (97) with pKD13, pKD46, and the primers P1 and P2. For construction of the Δ*fecR1*::*kan* mutation, the primers were designed to delete the +15 to +940 region (the first nucleotide in the initiation codon of the *fecR* gene was set to +1) to avoid the deletion of the stop codon of *fecI* overlapping with the start codon of *fecR* as well as the deletion of the promoter of *fecA* overlapping with the stop codon of *fecR* (38). YK627 and YK630 were constructed by transferring the Δ*fecR1*::*kan* marker from YK602 into MC4100 or YH426 by P1 transduction respectively, and the *kan* cassette of the resulting strain was deleted using pCP20. We confirmed by the assays with the *fec* reporter that, while the deletion of the *fecR* gene in YK627 (Δ*fecR1*) abolished the ferric citrate-dependent induction of transcription from P_{fecA} that was observed with a control *fecR*$^+$ strain, the expression of *fecR* from a plasmid fully restored the transcriptional activation of the *fec* operon (Fig. 3B). YK797 was constructed by transferring the Δ*rseP*::*kan* marker from KK377 into YK630 by P1 transduction.

Construction of plasmids

pKK47 (pTWV228 RseP) was constructed by cloning a 1.5 kb KpnI/HindIII fragment of pKK6 (10) into the same site of pTWV228. pYK2 (pTWV228 RseP(E23Q) was constructed by introducing the E23Q mutation into pKK47 by the standard site-directed mutagenesis method using appropriate primers. pSTD1060 (Spc^R vector) was constructed as follows. First, pHP45Ω (103) was digested with HindIII. Then a 2 kb HindIII fragment was blunt-ended by the T4 polymerase treatment. This fragment was ligated with pTWV228 that had been digested with ScaI and AseI and blunt-ended with T4 polymerase. In pSTD1060, the *aadA* (Spc^R) gene has been inserted in the same direction as the *bla* of pTWV228. pYK124 (pTWV228 FecR) was constructed as follows. A fragment containing

the complete ORF of *fecR* with the ideal SD sequence was amplified from chromosome of HM1742 by colony PCR using a pair of primers P3/P6. After digestion with SacI and HindIII, the fragment was cloned into the same site of pYH19. pYK186 (pSTD1060 FecR) was constructed by cloning a 1 kb EcoRI/HindIII fragment of pYK124 into the same site of pSTD1060. pYK126 (pTWV228 HA-FecR) was constructed as follows. A fragment containing the complete ORF of *fecR* except for the start codon was amplified from chromosome of HM1742 by colony PCR with primers P4/P6. Then, the fragment was digested with KpnI and HindIII and cloned into the same site of pYH19. To obtain pYK130 (pTWV228 3xFLAG-FecR), pYK126 was digested with SacI and KpnI and a resulting 4 kb fragment was ligated with the annealed oligonucleotides P7/P8. pYK188 (pSTD1060 3xFLAG-FecR) was constructed by cloning a 1 kb EcoRI/HindIII fragment of pYK130 into the same site of pSTD1060. pYK198 (pSTD1060 FecR85) and pYK200 (pSTD1060 3xFLAG-FecR 85) were constructed by replacing the codon for the Leu-86 of FecR on pYK124 or pYK130, respectively, with the ochre codon by site-directed mutagenesis and then cloning a 1 kb EcoRI/HindIII fragment of the resulting plasmids into the same site of pSTD1060. To obtain pYK143 (pTWV228 3xFLAG-MBP-RseA148), a 4 kb SacI/KpnI fragment of pYH19 was ligated with annealed polynucleotides P7/ P8. P7/P8 contains the sequence for the 3xFLAG tag with the ideal SD sequence. pYK147 (pTWV228 3xFLAG-MBP-FecR) was constructed as follows. A region encoding the transmembrane and periplasmic domains of FecR (from Asp-74 to the C-terminus) was amplified from the chromosome of HM1742 by colony PCR using primers P5/P6, digested with BamHI and HindIII and cloned into the same site of pYK143. pYK172, pYK170, pYK168, pYK166, pYK164, pYK174 and pYK162 (the C-terminally truncated mutant series of pTWV228 3xFLAG-MBP-FecR) were constructed by replacing the codons for the Arg-80, Leu-86, Thr-102, Leu-122, Ala-142, Ala-162 and Thr-182, respectively, of FecR on pYK147 with ochre codon by site-directed mutagenesis, and then a 0.7 kb BamHI/HindIII fragment of the resulting plasmids were cloned into the same site of pYK143. pYH604 (pTWV228-based constitutive expression vector) was constructed by the same procedure described previously (106). pYH604 had mutations in the *lacO1* operator on *lac* promoter regions of pTWV228 enabling the expression of a gene placed under the *lac* promoter in a constitutive manner. pYK182 (pYH604 3xFLAG-MBP-FecR) and pYK190 (pSTD1060 3xFLAG-MBP-FecR) were constructed by cloning a 2 kb EcoRI/HindIII fragment of pYK147 into the same site of pYH604 or pYK190, respectively. pYK140 (pTWV228 P_{fecA}) was constructed as follows. A promoter region of

fecA the -145 to -8 region with the the first nucleotide in the initiation codon of *fecA* set to +1) was amplified from chromosome of HM1742 by colony PCR using primers P9/P10, digested with KpnI and BamHI and cloned into the same site of pTWV228. The amplified fragment contains the -35 and -10 sequences in the *fecA* promoter also contains the Fur box (38). pYK145 (pFZY1 P_{fecA}-*lacZ*) was constructed by cloning a 0.2 kb EcoRI/HindIII fragment of pYK140 into the same site of pFZY1. pYK149 (pMW118 P_{fecA}-*lacZ*) was constructed by using In-Fusion HD Cloning Kit (Takara Bio) as follows. The P_{fecA}-*lacZ* region were amplified from pYK145 by PCR using primers P13/P14 and ligated with the vector fragment amplified from pMW118 with primers P11/P12. pYK151 (pSTD1060 RseP-His_6-Myc) and pYK153 (E23Q) were constructed by cloning a 1.5 kb SacI/HindIII fragment of pYH9 or pYH13, respectively, into the same site of pSTD1060.
